# Hyperthrombotic Milieu in COVID-19 Patients

**DOI:** 10.3390/cells9112392

**Published:** 2020-10-31

**Authors:** Mohamed Hassan Kamel, Wenqing Yin, Chris Zavaro, Jean M. Francis, Vipul C. Chitalia

**Affiliations:** 1 Renal Section, Department of Medicine, Boston University School of Medicine, Boston, MA 02118, USA; Mohamed.HassanKamel@bmc.org (M.H.K.); Wenqing.Yin@bmc.org (W.Y.); chriszavaro@gmail.com (C.Z.); jean.francis@bmc.org (J.M.F.); 2 Veterans Affairs Boston Healthcare System, Boston, MA 02132, USA; 3 Institute of Medical Engineering and Science, Massachusetts Institute of Technology, Cambridge, MA 02139, USA

**Keywords:** coronavirus, COVID-19, SARS-CoV2, respiratory failure, kidney failure, thrombosis, embolism, VTE, strokes, microvascular thrombosis, endotheliopathy, myocardial infarction

## Abstract

COVID-19 infection has protean systemic manifestations. Experience from previous coronavirus outbreaks, including the current SARS-CoV-2, has shown an augmented risk of thrombosis of both macrovasculature and microvasculature. The former involves both arterial and venous beds manifesting as stroke, acute coronary syndrome and venous thromboembolic events. The microvascular thrombosis is an underappreciated complication of SARS-CoV-2 infection with profound implications on the development of multisystem organ failure. The telltale signs of perpetual on-going coagulation and fibrinolytic cascades underscore the presence of diffuse endothelial damage in the patients with COVID-19. These parameters serve as strong predictors of mortality. While summarizing the alterations of various components of thrombosis in patients with COVID-19, this review points to the emerging evidence that implicates the prominent role of the extrinsic coagulation cascade in COVID-19-related coagulopathy. These mechanisms are triggered by widespread endothelial cell damage (endotheliopathy), the dominant driver of macro- and micro-vascular thrombosis in these patients. We also summarize other mediators of thrombosis, clinically relevant nuances such as the occurrence of thromboembolic events despite thromboprophylaxis (breakthrough thrombosis), current understanding of systemic anticoagulation therapy and its risk–benefit ratio. We conclude by emphasizing a need to probe COVID-19-specific mechanisms of thrombosis to develop better risk markers and safer therapeutic targets.

## 1. Introduction

### 1.1. Overview

Coronavirus disease 19 (COVID-19) is an acute viral illness caused by severe acute respiratory syndrome coronavirus 2 (SARS-CoV-2) and at the time of this report has resulted in a pandemic affecting people in 216 countries and territories [1]. First isolated from bronchoalveolar fluid in a Wuhan hospital [2], SARS-CoV-2 is the seventh member of the coronavirus (COV) family known to cause disease in humans [3,4,5]. This family of positive-sense single-stranded RNA viruses is divided into four genera [6,7], of which the alpha and beta subfamilies contain those relevant in human disease [6]. The beta-coronavirus genus, previously known to include two epidemic coronaviruses: severe acute respiratory syndrome (SARS-CoV-1) first identified in 2003 [5] and Middle East respiratory syndrome (MERS-CoV) identified in 2012 [4,8], now includes SARS-CoV-2 [2]. 

Thought to have zoonotic origins [9,10], the spike proteins of both SARS-CoV and SARS-CoV-2 binds to Neuropilin receptor, CD147/Basigin, heparin sulfate and CD209L/CD209, facilitating entry into the host cell [11,12,13,14,15]. Once infected, the human response to SARS-CoV-2 ranges from asymptomatic carriage to critical illness and death. Reported case fatality rates (CFR) have been variable in part due to denominator uncertainty and data lag [16].

With its relentless spread across the globe involving millions of people, the medical community has responded by identifying trends, generating hypotheses and trialing different therapeutic regimens. While the pandemic has affected almost all the aspects of daily life, the virus itself has demonstrated multi-organ system involvement [17]. Amongst the multitude of identified manifestations, abnormalities in coagulation and associated laboratory parameters were recognized early in COVID-19 patients [18] and shown to correlate with a poor prognosis [19]. This review explores what is known about the complex coagulation system and how it is impacted in COVID-19 with its translational implications.

### 1.2. Components of Hemostasis and Thrombosis

**Figure 1:**
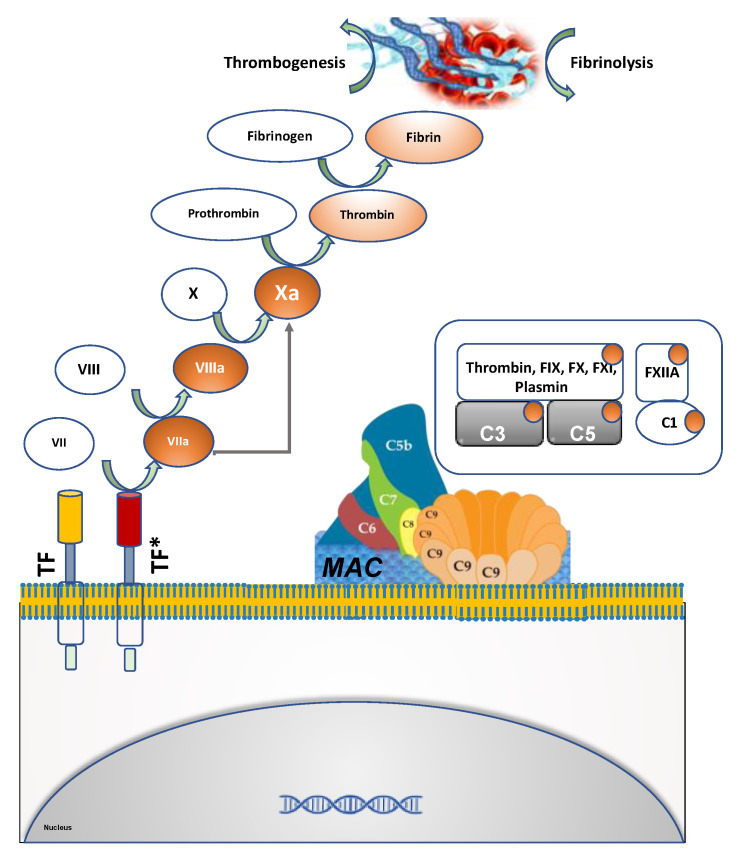
Extrinsic coagulation cascade is characterized by sequential activation and amplification of downstream components that finally culminate into the generation of the fibrin clot. Tissue factor (TF) is the primary trigger of the coagulation cascade. It is activated in the damaged endothelial cells. Platelets, polymorphonuclear cells and red blood cells (RBCs) entangled in the fibrin mesh result in clot expansion. Several components of the extrinsic coagulation cascade activate the complement system, as shown in the box.

Clotting is triggered by vascular injury and consists of several steps including activation of the coagulation cascade and formation of a platelet plug. Endothelial cells, polymorphonuclear cells and other components such as microparticles and complement system participate in this complex process. The coagulation cascade is traditionally divided into two pathways which converge to form fibrin, the final common pathway product that entangles platelets and other cellular elements to form and expand the clot. The intrinsic coagulation cascade (Figure 1), also known as the contact activation pathway characterized by initial activation of FXII, followed by sequential activation and amplification of FXI, followed by FIX activation, giving rise to the intrinsic coagulation cascade [20,21]. The extrinsic coagulation cascade, also known as the tissue factor pathway, is typically induced by trauma to tissue and endothelial cell activation. Tissue factor (TF) is the primary trigger of this pathway [22]. Under physiological conditions, vascular cells do not express high levels of TF. In pathologic conditions, such as endothelial cell damage or endothelial cell activation, TF expression is rapidly upregulated on the surface [23,24]. Both the intrinsic and extrinsic pathways converge to thrombin which converts fibrinogen to stabilize the fibrin clot, which rapidly entangles platelets leading to clot propagation. Clinically, changes in prothrombin time (PT) reflects alterations in the extrinsic coagulation cascade, while that in partial thromboplastin time (PTT) reflects alterations in the intrinsic coagulation cascade [25].

To restore normal blood flow, the clot must eventually be removed through a process called fibrinolysis [26]. Plasmin, a serine protease, breaks down the fibrin in the clot releasing fibrin degradation products (FDPs) such as D-dimer. Increased levels of FDPs is pathognomonic of ongoing coagulation and fibrinolysis. Both clotting and fibrinolysis are tightly regulated to prevent aberrant clot formation or bleeding diatheses. Uncontrolled activation of the coagulation cascade tilts the balance towards thrombogenesis [26]. In turn, this process can stimulate fibrinolysis and increased levels of FDPs in peripheral blood. 

The complement system is a key component of the innate immune response and cross talks with the coagulation cascade at several levels [27,28]. The complement system consists of approximately 30 proteins which upon exposure to specific triggers become sequentially activated, in turn enzymatically cleaving and activating the next protein in the cascade. Complement can be activated via three different pathways (classic, alternative and lectin), all of which culminate in the activation of C3. Activated C3 can trigger a lytic pathway by forming a multimeric protein complex, named the membrane attack complex (MAC), leading to cytolysis after inserting itself into the plasma membranes of affected cells. The clotting process can also activate the complement cascade. Several clotting and fibrinolytic components, such as thrombin, activated FIX, FX, FXI and plasmin, can cleave both C3 and C5, resulting in the activation of alternative pathways [29]. C5a can trigger the induction of TF expression and activity in human endothelial cells [30] and neutrophils [31]. Favoring a procoagulant state, C5a can also suppress the fibrinolytic activity of mast cells by inducing the expression of PAI-1 [32,33] and in turn augmenting clot progression. This extensive multi-level crosstalk between the coagulation and complement pathways is exploited by SARS-CoV-2 infection (vide infra).

### 1.3. Viral Infection, Inflammation and Thrombosis

Alterations in coagulation triggered by infections have been identified prior to the emergence of COVID-19. Under normal conditions, hemostasis is achieved by a balance of pro-coagulant and anticoagulant mechanisms [34]. This balance can be tipped in favor of coagulation by inflammation accompanying infection, a concept which has been well established in several infectious agents [35,36,37]. Studies in ancient organisms such as the horseshoe crab have revealed coagulation and immune systems that are deeply integrated, and when activated work in concert to trap pathogens in an insoluble coagulin medium [38]. In humans, these two systems continue to be closely interlinked with crosstalk on multiple levels [39,40,41].

Viral pathogens disrupt the coagulation cascade at various levels to augment bleeding diathesis and augment thrombosis. Viruses including Ebola, Marburg, Dengue, Huaiyangshan virus and Crimean–Congo hemorrhagic fever (CCHF) give rise to hemorrhagic fever in the host via multiple mechanisms, including cytokine-mediated endothelial activation and damage, clotting factor consumption and direct hepatic toxicity causing decreased production of clotting factors [42]. Hemorrhagic complications have rarely been noted in infections with non-hemorrhagic viruses such as varicella zoster virus (VZV) [42,43,44,45], cytomegalovirus (CMV) [46,47,48] and Epstein-Barr virus (EBV) [49]. On the other end of the spectrum, thrombotic complications including deep vein thrombosis (DVT), pulmonary embolism (PE), thrombotic microangiopathy (TMA), thrombotic thrombocytopenic purpura (TTP) and portal vein thrombosis have been reported in many viral infections including Avian influenza (H5N1), Swine flu (H1N1), Parvovirus B19, CMV, EBV, VZV, Hepatitis A and Hepatitis C virus and human immunodeficiency virus (HIV) [50]. 

The mechanisms behind these thrombotic manifestations are not fully understood in part due to the complex interplay of host, virus and environmental factors. For instance, CMV-associated thrombosis has been demonstrated in hosts with underlying thromophilic predispositions such as factor V Leiden mutation [51], protein C and S deficiency [52] and anti-phospholipid syndrome [53]. Viral infections have demonstrated several unique mechanisms in propagating thrombosis. For example, in a murine model, injection of a synthetic CMV peptide induced anti-β2GPI antibodies which resulted in the activation of endothelial cells and enhanced thrombus formation [54]. While thromboembolic events and responsible mechanisms have been elucidated in many viral infections, SARS-CoV-2 seemingly differs in incidence and potential mechanisms. In the following section, we discuss both the microvascular and macrovascular thrombosis in the arterial and venous systems of patients affected by COVID19.

## 2. Arterial Thrombosis

Increased thrombotic events are reported in SARS-CoV-2 and SARS-CoV-1. One report noted large artery strokes in 5 out of 206 patients hospitalized with SARS-CoV-1 despite therapeutic anticoagulation and relatively fewer vascular risk factors [54]. The investigators concluded that the SARS-CoV-1 acute infection induced a hypercoagulable state which along with hypotension and cardiac dysfunction gave rise to cerebral arterial thromboembolism.

In a series of 184 Dutch patients critically ill with COVID-19, 7 developed arterial thrombotic events, 5 of which were ischemic strokes and the other 2 were systemic arterial embolisms, all despite standard dose thromboprophylaxis [55]. In a larger series in Italy, 13 out of 362 patients developed evidence of arterial thrombosis manifesting as stroke and myocardial infarction, half of which were identified within 24 h of presentation to the hospital [56]. Disease of the aorta has also been reported in two patients seemingly without predisposition, with one patient developing occlusion of the infrarenal aorta and common iliac arteries, and the other an occluded descending thoracic aorta with embolism to the superior mesenteric artery [57]. Superior mesenteric artery thrombosis was diagnosed in a COVID-19 patient in whom anticoagulation was withdrawn [58]. Lower extremity thrombosis has also been described in two patients developing popliteal artery thrombosis (one of which with concomitant CVA), in spite of therapeutic dose anticoagulation (enoxaparin in one case and argatroban in the other) [59]. Collectively, the above reports underpin the augmented incidence of thrombosis in several arterial beds in patients despite a lower risk profile for thrombosis and thromboprophylaxis therapy.

## 3. Venous Thrombosis and Pulmonary Embolus

Perhaps one of the earlier manifestations of perturbed coagulation cascade identified in relation to COVID-19 was elevation of D-dimer, a fibrin degradation product [60]. This is a marker of coagulation cascade activation in the microvascular beds which has been shown to be pathologically elevated in 46% of SARS-CoV-2-infected patients and in 56% of those with severe disease [61]. In fact, D-dimer levels > 1 μg/mL have been shown to be directly related to COVID-19 in hospital mortality [62]. In comparison to survivors, patients who did not survive had on average a three-fold increase in D-dimer levels [19]. In addition, D-dimer served as the surrogate marker for the thrombotic burden, supported by the finding of elevated D-dimer levels in nearly all cases of venous thromboembolism (VTE) [63,64,65,66,67] in the setting of COVID-19 infection.

Initial reports from China demonstrated a VTE rate of 25% in patients admitted to the intensive care unit (ICU) with COVID-19—none of whom were on prophylactic dose anticoagulation [68]. Additional reports have demonstrated that patients with acute pulmonary embolism had higher D-dimer levels [69]. In one ICU cohort of 184 patients, the cumulative incidence of VTE was 27 (15% of total), despite all receiving prophylactic dose anticoagulation [55,70]. Age and abnormal coagulation parameters (prothtrombin time (PT) and activated partial thromboplastin time (aPTT) prolongation) were identified as independent predictors of thrombosis. An additional 40 VTE events were reported upon follow-up of these patients for an additional two weeks [55,70]. Similarly, a prospective cohort compromised of ICU patients admitted with COVID-19-induced acute respiratory distress syndrome (ARDS) demonstrated an 18% incidence of VTE, a value significantly higher than the incidence in a matched non-COVID ARDS cohort [71].

Strikingly high rates of VTE have been reported in severe COVID-19 infection despite anticoagulation, with one study reporting an overall rate of 69% when all patients were screened regardless of symptoms or clinical suspicion [72]. This pattern was again replicated in another cohort of 107 patients admitted to the ICU with COVID-19, in which 20% were found to have PE despite nearly all patients receiving thromboprophylaxis. It is noteworthy that when this cohort was compared to a similar one hospitalized a year earlier and a smaller influenza cohort from the prior season, the frequency of PE was twice as high in the COVID group despite less computed tomography (CT) angiogram tests performed [73]. In a less critically ill cohort, a 4% VTE rate was observed, which was considered to be an underestimate [56]. In an autopsy series of 12 subjects, 7 were found to have venous thromboembolism that was not suspected prior to death, and 4 patients were diagnosed as having a terminal PE [74]. Mirroring this finding, another autopsy series of 10 patients revealed small pulmonary artery fibrinous thrombi in 8 patients [75]. Taken together, these studies implicate COVID-19 to be a highly thrombogenic milieu increasing the risk of both arterial and venous thrombosis.

Emerging evidence suggest influence of racial predilections in thromboembolic events in COVID19 patients. In general, African Americans are at a higher risk of thromboembolic events [76,77]. Recent trends from several areas in the United States have shown that greater than 50% of COVID-19 cases and 70% of COVID-19-related deaths were in African Americans, even though they make up only 30% of the study population [78,79,80,81]. While several factors such as healthcare disparities, socioeconomic status and comorbid conditions may play a role in this trend, studies have shown evidence of severe pulmonary and cardiac pathologies along with increased thrombosis in African Americans with severe COVID-19 compared to other racial groups [82,83]. More research is needed to deeply understand the influence of race on COVID19 and thromboembolic events.

## 4. Microthrombi in Vascular Beds

The catastrophic end organ damage and multisystem organ failure associated with COVID-19 may stem from abnormalities in microvascular beds. An early clue into the possible involvement of the microvascular bed arose from the observation that COVID-19 patients presented with profound hypoxia which was out of proportion to the preserved lung mechanics suggestive of significant pulmonary shunting [84], raising the possibility of a lung injury mechanism different from that of traditional ARDS [69]. Similarly, multi-organ dysfunction was noted to be out of proportion to the degree of sepsis and shock, all pointing to the presence of systemic vasculopathy and involvement of microvasculature.

The widespread presence of microvascular thrombosis was demonstrated in autopsy studies. In one autopsy series, 5 out of 11 COVID-19 subjects showed alveolar capillary microthrombi and 3 out of 18 showed glomerular capillary microthrombi, despite receiving anticoagulation [85]. A common theme of microvasculature thrombosis of lung parenchyma was also demonstrated in a smaller autopsy series [86]. Ackerman et al. compared lungs of 7 patients who died from COVID-19 to 7 lungs of patients who died of influenza A(H1N1)-induced ARDS, observing widespread thrombosis with microangiopathy [87]. Analysis of precapillary vessels showed the presence of thrombi in the pulmonary arteries of 4 out of the 7 lungs from patients with COVID-19. The alveolar capillary microthrombi were 9 times as prevalent in patients with COVID-19 as in patients with influenza patients [87]. In summary, microvascular thrombosis is an important contributor to end organ dysfunction such as respiratory failure and acute kidney injury, both important drivers of mortality.

## 5. COVID-19-Induced Thrombosis

### 5.1. Characteristics of Coagulopathy in COVID-19 Patients

Several studies have demonstrated the coagulation abnormalities in COVID-19 patients to be characterized as [88,89] (a) elevation of FDPs such as D-Dimer, (b) modestly elevated PT, (c) mostly normal or slightly depressed PTT, (d) variable variations in platelet counts, (e) elevated levels of fibrinogen, von Willebrand factor and factor VIII levels and (f) normal levels of factor XIa. This constellation of parameters indicates an activation of extrinsic coagulation cascade with little activation of the intrinsic coagulation pathway.

The clinical relevance of these coagulation abnormalities in COVID-19 was realized quite early in the course of the pandemic [19,90,91]. The presence of coagulopathy was associated with strikingly increased mortality rates [19]. Furthermore, the degree of alteration in these parameters correlated with the severity of illness. Higher median level of D-dimer was predictive of ICU admission [92], and preciously heightened (18-fold) risk of death above levels of 1 μg/mL [62]. PT prolongation was more likely to be detected in COVID-19 non-survivors [19]. In addition, thrombocytopenia was also found to be associated with increased severity of illness by over 5-fold [93]. Elevated levels of fibrinogen were identified in COVID-19 patients in comparison to controls, and a subgroup with higher levels was more likely to develop respiratory distress [94]. All these observations indicate strong association of coagulopathy with the overall mortality and organ failure in COVID-19 patients.

The endothelium has complex functions and when disturbed can shift the balance towards vasoconstriction, thrombosis and inflammation [95]. Normally, the endothelial layer consists of quiescent endothelial cells, which are anti-inflammatory and anticoagulant while expressing lower levels of tissue factor activity, the primary stimulant of the extrinsic coagulation cascade [23,24]. These endothelial cells prevent inflammation by inhibiting their interaction with immune cells and platelets and by expressing coagulation inhibitors and fibrinolytic enzymes. Endothelial cells also produce a glycocalyx, a protective layer of glycoproteins and glycolipids with anticoagulation properties [96]. With endothelial cell damage, activity or expression of surface TF increases. This in turn facilitates the conversion of Factor VII to VIIa, activating a cascade that culminates in fibrin generation.

Emerging evidence implicates the underlying presence of endothelial damage (endotheliopathy) in patients with severe COVID-19 [97,98]. An autopsy examination of lungs of COVID-19 patients using micro-computed tomographic imaging, scanning electron microscopy and corrosion casting showed severe endothelial injury associated with the presence of intracellular virus and disrupted cell membranes [87]. Goshua et al. examined the blood markers of endothelial cell damage (VWF antigen and thrombomodulin) and platelet activation (soluble P-selectin derived from Weibel-Palade bodies) in 68 patients with COVID-19, comparing 48 ICU patients to 20 non-ICU patients, and 13 non-hospitalized and asymptomatic controls [88]. The markers of endothelial cell and platelet activation were significantly elevated in ICU patients compared with non-ICU patients. Mortality significantly correlated with elevated VWF antigen and soluble thrombomodulin. Elevated plasma VWF concentrations are highly suggestive of fulminant endothelial cell activation and robust platelet activation [97].

COVID-19 patients exhibit alterations consistent with on-going intravascular coagulation and fibrinolysis processes (reflected by increased FDP), a condition similar to disseminated intravascular coagulation (DIC). DIC is a catastrophic hematological condition defined by consumptive coagulopathy [99] and the concurrent presence of microthrombosis and bleeding diathesis. The hematological characteristics of COVID-19-associated DIC seem to differ from traditional sepsis-induced DIC. COVID-19-associated DIC is uniquely characterized by profound elevation of D-dimer and mild thrombocytopenia, in comparison to traditional sepsis-related DIC which is typically characterized by severe thrombocytopenia and lower fibrinogen levels [91]. In addition, hemorrhagic complications secondary to COVID-19-induced coagulopathy are not frequently reported [100].

### 5.2. SARS-CoV-2 and Coagulopathy

**Figure 2:**
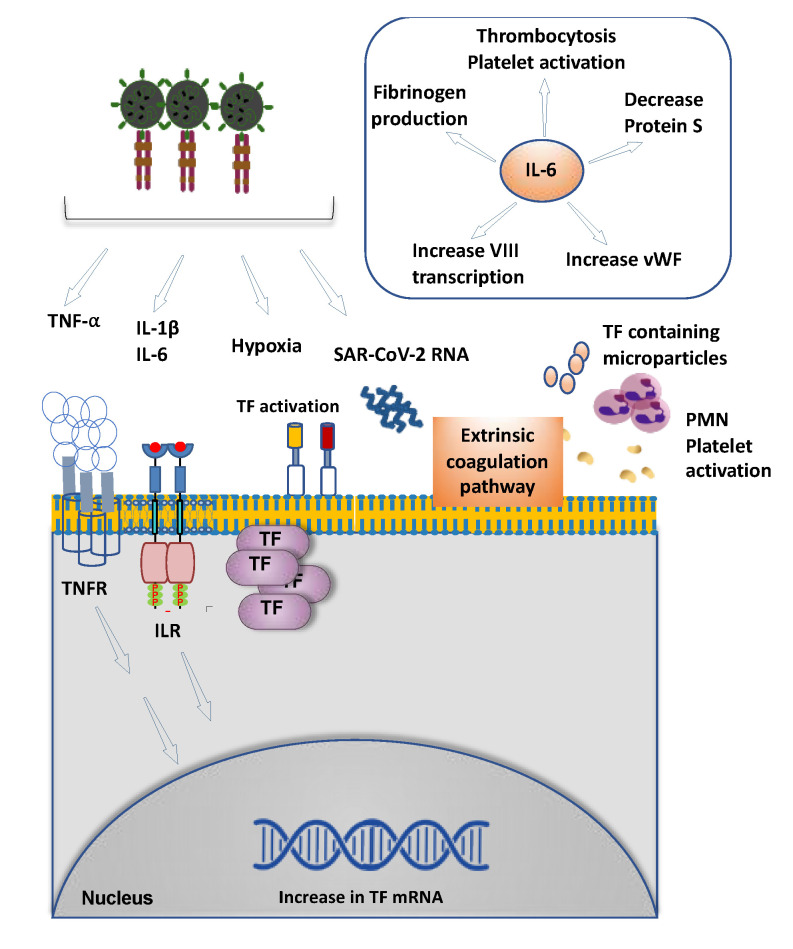
The prothrombotic milieu in COVID-19 patients is generated by several factors of which stimulation of extrinsic coagulation assumes a central stage. Damage to the endothelial cells activates TF which serves as the primary trigger for the extrinsic coagulation cascade. Several components of cytokine storm such as tumor necrosis factor alpha (TNF-α), interleukin-1 β (IL-1β) and interleukin-6 (IL-6) (through their cognate receptors and hypoxia associated with shock) upregulate TF mRNA in the endothelial cells. Other cells involved in the thrombotic process such as platelets and polymorphonuclear cells (PMNs) are activated in the milieu of cytokine storm and secrete TF-laden microparticles that augment thrombogenesis. SARS-CoV-2 RNA in the blood can serve as an activator of extrinsic coagulation cascade. (Insert) IL-6 augments thrombosis by several mechanisms. IL-6 increases the secretion of prothrombotic von Willerbrand facror (VWF) and factor VIII from liver and induces thrombocytosis in bone marrow. It also downregulates protein S in blood, a known inhibitor of thrombogenesis.

SARS-CoV-2 can trigger this aberrant thrombotic process directly or indirectly (Figure 2). It is conceivable that after viral entry into cells, viral proteins directly interact with the host proteins involved in the coagulation cascade. This scenario is plausible since the entry of virus into endothelial cells expressing the receptors for SARS-CoV-2 including Angiotensin-converting enzyme 2 (ACE2) and proteases such as Transmembrane protease, serine 2 (TMPRSS2), has been demonstrated [98]. SARS-CoV-2 viral elements are observed within endothelial cells by several investigators [87,101]. It is plausible that once inside the cells, the viral particles or specific protein can interact or alter the activity of proteins such as TF directly or indirectly through signaling events. While a detailed map of the interaction of various SARS-CoV-2 viral proteins with host proteins has been described [102], to date, no direct interaction of viral proteins with the proteins of the coagulation cascade has been demonstrated. In a mice model of arterial thrombosis, extracellular SARS-CoV-2 RNA was found to be associated with fibrin-rich thrombi, but significantly reduced with pretreatment of ribonuclease [103]. It is plausible that SARS-CoV-2 RNA derived from damaged or necrotic cells in conditions of severe tissue damage activates the factors XII/XI-mediated contact activation pathway.

Infection of various cells including endothelial cells with SARS-CoV-2 can indirectly stimulate coagulation. SARS-CoV-2 infection can induce a cytokine storm characterized by upregulation of IL-6 and IL-1, etc. [104,105,106,107], and this observation constitutes the rationale of anti-IL-6 and anti-IL-1 therapeutics in COVID-19 patients. Patients infected with SARS-CoV-2 admitted to intensive care units (ICUs) have elevated serum levels of IL-1β, IL-2, IL-6, IL-7, IL-10, macrophage colony-stimulating factor (M-CSF), granulocyte colony-stimulating factor (G-CSF), granulocyte-macrophage colony stimulating factor (GM-CSF), interferon-gamma-induced protein (IP-10), monocyte chemoattractant protein-1 (MCP-1), macrophage inflammatory protein 1-α (MIP 1-α) and TNF-α [104,105,106,107].

Several of these cytokines augment TF expression and activity in different cells important in thrombosis, including endothelial cells, monocytes and pericytes, etc. [23,24]. Induction of TF primarily occurs at the transcriptional level, resulting in an increase in TF mRNA and eventually, TF protein surface expression. Moreover, TF-containing microparticles are released from the cell. TNF-α increases TF RNA through TNF-receptor, similarly, vascular endothelial growth factor (VEGF), thrombin and IL-1β signal through kinase insert domain receptor (KDR), protease activated receptors (PARs) and IL-1R to increase TF mRNA. In addition, SARS-CoV infection-induced severe hypoxia and associated ischemic tissue injury can induce or release the TF from dysfunctional endothelial cells, monocytes, platelets and chemotaxis neutrophils [23,108,109].

Wan et al. detected elevated IL-6 levels in one-third of patients with mild symptoms and three-quarters of those with severe symptoms, suggesting that IL-6, alongside IL-10, may be of prognostic value in patients with COVID-19 [110]. IL-6 has assumed a central position in the COVID-19-related cytokine storm. It has been shown that IL-6 promotes coagulation [111]. IL-6 promotes thrombosis through a number of pathways [112]. IL-6 increases TF production by monocytes and factor VIII transcription in hepatocytes and the serum levels of VWF, while suppressing Protein S, an inhibitor of thrombosis. Also, IL-6 increases fibrinogen production by a variety of cells as well as increasing platelet production and enhancing platelet activation.

There is a complex interaction between the coagulation pathway and the cytokine network [113] (Figure 2). Activation of the TF pathway can exacerbate cytokine storm and vice versa. TF is the high-affinity receptor and cofactor for factor VII/VIIa, which can increase the concentrations of IL-6 and IL-8 in human plasma [114]. An inhibitor of TF—factor VIIa abrogated this increase in IL-6 and IL-8. Collectively, the literature supports potential mechanisms of induction of thrombosis by SARS-CoV-2 infection, however, several of them warrant direct proof in the context of COVID-19 infection.

### 5.3. Complement Cascade and Thrombosis

Studies have demonstrated an activated complement cascade in various tissues of COVID-19 patients [115,116]. A series of cutaneous biopsy and pulmonary autopsy samples from 5 patients with severe COVID-19 revealed a pattern of TMA and severe activation of the complement cascade as evidenced by vascular deposits of C5b-9 (membrane attack complex), mannose-binding protein-associated serine protease 2 (MASP2) and C4b, demonstrating severe activation of the complement cascade [117]. Co-localization of SARS-CoV-2-specific spike glycoproteins with complement components has been demonstrated in the lung, skin and kidney tissue. The activation of complement is also observed in the experimental models of SARS-CoV-2 and linked to the number of neutrophils. A mouse model with SARS-CoV-2 infection showed early activation of complement cascade [118]. SARS-CoV-2-infected C3 genomic knock-out mice demonstrated lower numbers of neutrophils [118]. 

Activation of complement in COVID-19 patients can trigger thrombosis in microcirculation. For example, C5a can directly activate the TF pathway and extrinsic coagulation cascade [31]. Also, C5a can upregulate the expression of plasminogen activator-inhibitor-1 in human mast cells and basophils, thereby inhibiting fibrinolysis [32]. Anti-C5a therapeutics ameliorated coagulation and fibrinolysis in a rat model of sepsis [119], suggesting the critical role of complement in effecting the balance between procoagulant response and anticoagulation cascade. Using peripheral blood mononuclear cells (PBMC) from COVID-19 patients, Li et al. have shown an interaction of viral protein nsp9/nsp10 with C1Q binding protein (C1QBP) [120]. This event likely leads to the activation of C1QBP which is required for the activation of complement C1 in the classical pathway. Gao et al. identified the host complement activator MASP2 as a target of the N protein of SARS-CoV-1, SARS-CoV-2 and MERS-CoV viruses [121]. In mice, lung injury induced by SARS-CoV-1 or MERS-CoV N protein was attenuated when its MASP2-binding motif was altered, in MASP2 -/- mice, or when the MASP2–N protein interaction was pharmacologically blocked. A link between complement activation and organ failure in patients with COVID-19 is suggested by the preliminary data of potential benefit of targeting complement in patients with COVID-19 using a blocking antibody to complement components [122,123]. In summary, activation of an alternative pathway and MBL complement pathway in COVID-19 patients can be trigged through various mechanisms. Complement activation by viral proteins either via the activation of the MBL pathway or the alternate pathway (AP) leads to endothelial injury and C5a release that triggers massive inflammation and microthrombi formation, giving rise to catastrophic manifestations in the lungs [117], amongst other organs.

### 5.4. Antiphospholipid Antibodies

Recent publications have brought to light anti-phospholipid antibodies in COVID-19 patients as a potential piece of the thrombotic puzzle. This family of antibodies target phospholipid-binding proteins on various surfaces and include anti-β2-glycoprotein I (anti-β2GPI), lupus anticoagulant (LA) and anti-cardiolipin (aCL) [124]. These antibodies constitute the anti-phospholipid syndrome, which is characterized by thrombotic events that require life-long anticoagulation [125]. In a French study, 50 out of 57 critically ill patients tested positive for LA [71]. In another study, 25 out of 56 were found positive for a LA [126]. One patient with ischemia of both lower limbs, digital ischemia of one upper limb and bilateral ischemic strokes tested positive for aCL IgA and anti-β2GPI IgA and IgG antibodies. Two other patients were reported to have similar findings [127]. A trend towards higher titers of antiphospholipid antibodies is also observed in several other viruses, including HIV, CMV and EBV. In most instances, these antibodies were transient, but in others, they seemed to trigger the onset of full-blown anti-phospholipid syndrome [128,129,130]. 

### 5.5. Platelets and WBCs

Platelets are a critical component of thrombosis. Although thrombocytopenia is common in close to 40% of patients with COVID-19 [131,132], several structural and functional abnormalities that enhance thrombogenicity have been observed. COVID-19 patients showed platelets with larger mean platelet volume, which is known to increase the risk of thrombosis as they tend to bind more fibrinogen than smaller platelets [133]. In general, less than 5% of human unstimulated platelets show p-selectin positivity. In contrast, patients with COVID-19 showed higher levels of p-selectin, CD63 and tissue factor expression both at baseline and upon activation by thrombin receptor-activating peptide (TRAP) [134,135]. Platelets from COVID-19 patients showed greater aggregation in response to several platelet agonists [136] and displayed greater propensity for interactions with neutrophils, monocytes and T cells compared with healthy donors. Also, they demonstrated greater spreading on fibrinogen and collagen through upregulation of the mitogen-associated protein kinases (MAPK) pathway, and increased thromboxane generation when compared to controls [136]. One study showed viscoelastic properties of platelets that augmented clot strength even in COVID-19 patients with normal platelet counts [137]. 

Several potential mediators of platelet activation exist in COVID-19 patients including cytokines (IL-6, etc.), chemokines and profound endothelial damage, etc. Interestingly, recent evidence has demonstrated expression of ACE2 receptors and TMPRSS2 on platelet surface that can allow direct viral entry in platelets [138]. Increased ACE2 receptors and TMPRSS2 expression was driven by stimulatory effects of SARS-CoV-2 or its spike protein. In support, Zhang et al. have recently shown that the recombinant human ACE2 protein or anti-spike monoclonal antibody to inhibit spike protein-induced platelet activation [138]. Overall, these qualitative and functional alterations in platelets contribute to the hyperthrombotic milieu in COVID-19.

Emerging evidence point to monocytes and neutrophils contributing to the hyperthrombotic milieu in COVID-19 patients. Neutrophils are a part of the innate immunity and induce the formation of Neutrophil Extracellular Traps (NETs). In general, NETs are composed of nuclear chromatin, nuclear histones, microvesicles and granular antimicrobial proteins that are meant to trap and kill pathogens [139]. Middleton et al. [140] studied the connection between NETs and severity and progression of COVID-19 both in a prospective cohort of patients and in autopsy studies. Soluble and cellular factors triggering NETs were significantly increased in COVID-19 patients. The autopsy studies confirmed NET-containing microthrombi within pulmonary vasculature. Neutrophils from COVID-19 patients displayed excessive NETs at baseline, and COVID-19 plasma triggered NET formation, which was blocked by neonatal NET-inhibitory factor. Skendros et al. [141] examined interplay of NETosis and complement activation. Their results demonstrated that the treatment of control neutrophils with COVID-19 platelet-rich plasma generated TF-bearing NETs, inducing thrombotic activity of endothelial cells. Plasma from COVID-19 patients demonstrated increased tissue factor (TF) activity and sC5b-9 levels. Neutrophils of COVID-19 patients yielded high TF expression and released NETs carrying active TF. Inhibition of both NETosis or C5aR1 was required to attenuate platelet activation and NET-driven thrombogenicity. Complement C3 inhibition with compstatin Cp40 disrupted TF expression in neutrophils. Taken together, these studies uncover complex interactions between innate immunity, complement system and thrombosis (immunothrombosis) in COVID-19 patients [142].

## 6. Therapeutic Considerations

All confirmed or presumed COVID-19 patients admitted to the hospital undergo close monitoring for the risk of thrombosis. A panel of coagulation cascade is performed on admission and repeated during hospitalization. This panel includes: D-Dimer, PT, aPTT, fibrinogen and platelet count. 

### 6.1. VTE Prophylaxis

All confirmed or presumed COVID-19 patients admitted to the hospital usually receive prophylactic anticoagulation to prevent VTE, unless contraindicated. Low molecular weight heparin (LMWH) or unfractionated heparin (UFH) can be used. Many reports have suggested that ICU patients with COVID-19 infection remain at a higher risk of VTE despite standard anticoagulation prophylaxis [55,70,71]. Data from these reports suggest the need for higher prophylactic doses of LMWH (e.g., enoxaparin 0.5 mg/kg twice daily) for VTE prevention in COVID-19 patients, in comparison to what is conventionally used for non-COVID-19 ICU patients. One can conceive a safer prophylactic strategy guided by the levels of D-Dimer, fibrinogen, or other inflammatory markers [143,144]. Every effort should be made to avoid missing prophylactic anticoagulation dosage as this has been associated with worse outcomes [145]. In clinical scenarios where thromboprophylaxis is contraindicated, intermittent pneumatic compression should be used in immobilized COVID-19 patients. Several questions remain unanswered, including optimum dose of anticoagulation and duration of prophylactic anticoagulation after discharge from the hospital, etc. Importantly, the benefits of prophylactic anticoagulation should always be weighed against the risks of bleeding, a serious threat in some acutely ill COVID-19 patients [146,147].

### 6.2. Prophylactic Anticoagulation in COVID-19 Patients

In line with the high rates of thrombotic events in patients with severe COVID-19, therapeutic-dose anticoagulation is given consideration even in the absence of documented thrombosis. A widely quoted study supporting this approach was performed by Paranjpe et al. who reported an association between therapeutic anticoagulation and in-hospital survival among mechanically ventilated patients with COVID-19 [148]. This was a retrospective cohort study where 28% of 2773 patients with COVID-19 received some form of systemic anticoagulation during their hospital stay. Mortality was nearly identical among patients with therapeutic anticoagulation (22.5%) compared to the controls who did not receive it (22.8%). Patients who received therapeutic anticoagulation were more likely to require invasive mechanical ventilation than the control (30% vs. 8%). In-hospital mortality was 63% (median survival, 9 days) among mechanically ventilated patients who did not receive anticoagulation and 29% (median survival, 21 days) among those who received therapeutic anticoagulation. 

This study had several limitations. For example, anticoagulation was administered in this study based on a clinical indication. Their analysis did not report confounders. The most serious flaw of the study is immortal time bias. Immortal time is the interval in an observational study between the time a patient enters a cohort and the time they receive the intervention in question. During the interval between admission and anticoagulation, death is not considered in the anticoagulation group because those patients had to survive long enough to receive treatment. This implies that the deaths that occurred during immortal time could be attributed only to the “no anticoagulation” group, thus biasing the results in favor of anticoagulation. However, this retrospective study has stimulated many randomized, controlled trials of anticoagulation strategies which are underway.

### 6.3. Choice of Anticoagulant Agents

Treatment of established VTE or arterial thrombosis in the setting of COVID-19 remains a challenging issue. The therapy of thromboembolic events in COVID-19 patients is extrapolated from the previous experience with other forms of sepsis-induced coagulopathy (SIC). Other potential therapeutic considerations include physiologic anticoagulants like activated protein C, thrombomodulin and antithrombin. It is important to note that levels of antithrombin have not been shown to be significantly reduced in COVID-19 patients, raising questions regarding the benefits of antithrombin therapy [19,149]. These medications have been used in the treatment of SIC with anecdotal evidence when associated with DIC [150,151,152]. These reports may support the use of those drugs in a very selected group of COVID-19 patients with a predominant DIC picture. 

Unfractionated heparin (UFH) remains the mainstay of therapy in those circumstances. UFH is commonly used due to its short half-life and ease of anticoagulation effect reversal in case of bleeding or the need to perform any procedures. Low molecular weight heparins (LMWHs) are another excellent alternative especially if no invasive procedures are planned. Direct oral anticoagulants (DOAC) such as apixaban (Eliquis), etrixaban (BevyxXa), dabigatran (Pradaxa), edoxaban (Savaysa) and rivaroxaban (Xarelto) can also be used with the advantage of no need for therapeutic monitoring. Reversal of bleeding in the setting of DOACs might not be widely and timely available. 

Many recent reports have highlighted the role of the complement system in the pathophysiology of severe COVID-19 respiratory and multiorgan failure [117,153,154]. This has led to trials that are actively recruiting patients to study the benefits of eculizumab, a C5 inhibitor, in patients with severe respiratory failure from COVID-19 (Clinical trial NCT04288713 and NCT04346797). 

Currently, data regarding bleeding risk in the setting of COVID-19 is limited. This aspect is critical to examine since patients with COVID-19 are on thromboprophylaxis and therapeutic anticoagulation at times with co-existing thrombocytopenia, all of which increase the risk of bleeding [155]. With lack of clear evidence of bleeding tendencies in most patients receiving thromboprophylaxis and therapeutic anticoagulation, most centers exercise caution in managing COVID-19 patients who are at increased risk of bleeding from other causes such as immune thrombocytopenia and hemophilia, and those with history of a prior major bleeding episode. If bleeding occurs, the treatment is similar to non-COVID-19 patients and includes blood transfusions, anticoagulant reversal or discontinuation, or specific products that address underlying bleeding disorders.

## 7. Conclusions

Thromboembolism drives multisystem organ failure and fatal complications of SARS-CoV-2. SARS-CoV-2 triggers an array of mechanisms that culminate in the propagation of a hyperthrombotic milieu. Several aspects of such mechanism remain elusive. Profound alterations in coagulation parameters, their association with overall mortality and early signs of clinical benefit with systemic anticoagulation all strongly indicate the pivotal role of thrombosis in patients with COVID-19. Clinical predictors tools to accurately stratify COVID-19 patients at risk of thromboembolic event are limited to a handful of tests. The therapeutic landscape of hyperthrombotic milieu in COVID-19 also remains challenging with breakthrough thrombosis despite sufficient thromboprophylaxis or therapeutic anticoagulation. While agents such as activated protein C, PAI-1 antagonists and tissue plasminogen activators (tPA) are proposed, these investigational agents and food and drug administration(FDA)-approved antithrombotics target fundamental machinery of hemostasis, thereby enhancing the potential for bleeding in this sick cohort of patients. All these points represent areas of unmet clinical need and warrant further investigation in the therapeutics targeting SARS-CoV-2-induced mechanisms of thromboembolic events in COVID-19 patients.
